# More than a checklist: a realist evaluation of supervision of mid-level health workers in rural Guatemala

**DOI:** 10.1186/1472-6963-14-112

**Published:** 2014-03-06

**Authors:** Alison R Hernández, Anna-Karin Hurtig, Kjerstin Dahlblom, Miguel San Sebastián

**Affiliations:** 1 Umeå International School of Public Health, Department of Public Health and Clinical Medicine, Umeå University, 901 87 Umeå, Sweden

**Keywords:** Mid-level health worker, Supervision, Performance, Motivation, Realist evaluation, Guatemala

## Abstract

**Background:**

Mid-level health workers (MLHWs) form the front-line of service delivery in many low- and middle-income countries. Supervision is a critical institutional intervention linking their work to the health system, and it consists of activities intended to support health workers’ motivation and enable them to perform. However its impact depends not only on the frequency of these activities but also *how* they are carried out and received. This study aims to deepen understanding of the mechanisms through which supervision activities support the performance of auxiliary nurses, a cadre of MLHWs, in rural Guatemala.

**Methods:**

A multiple case study was conducted to examine the operation of supervision of five health posts using a realist evaluation approach. A program theory was formulated describing local understanding of how supervision activities are intended to work. Data was collected through interviews and document review to test the theory. Analysis focused on comparison of activities, outcomes, mechanisms and the influence of context across cases, leading to revision of the program theory.

**Results:**

The supervisor’s orientation was identified as the main mechanism contributing to variation observed in activities and their outcomes. Managerial control was the dominant orientation, reflecting the influence of standardized performance criteria and institutional culture. Humanized support was present in one case where the auxiliary nurse was motivated by the sense that the full scope of her work was valued. This orientation reflected the supervisor’s integration of her professional identity as a nurse.

**Conclusions:**

The nature of the support health workers received was shaped by supervisors’ orientation, and in this study, nursing principles were central to humanized support. Efforts to strengthen the support that supervision provides to MLHWs should promote professional ethos as a means of developing shared performance goals and orient supervisors to a more holistic view of the health worker and their work.

## Background

Mid-level health workers (MLHWs) are the point of contact between the health system and the most vulnerable populations in many low and middle income countries. Their performance is a key leverage point for efforts to redress inequities and enable achievement of the highest attainable standard of health. MLHWs are health care providers with formal accreditation but they have shorter training and a more restricted scope of practice than professionals [1]. In many low and middle income countries, they constitute the mainstay of the health workforce because compared to health professionals they are easier to attract and retain in underserved areas and cheaper to train and deploy. In this way MLHWs contribute to increased access to services. But in order to improve their performance, greater attention to how the health system operates to support them is needed [2].

Supervision is a common institutional intervention in health systems intended to support health workers’ capacity and motivation to perform. It provides a link between service delivery in peripheral units and district management through measures to ensure that health workers carry out their work effectively and enable them to improve their competence [3]. Supervision serves as the point of human interconnection between health workers and the health system and it can encourage motivation through orientation to the organization’s values [4]. This contact is a central tool for supporting front-line health workers such as MLHWs who deliver care in isolated rural settings with limited training [5].

In order to contribute to performance, international recommendations emphasize that supervision should take a supportive approach that promotes quality through strengthened relationships, identification and resolution of problems and constructive feedback [6,7]. However, these recommendations are not easily translated to practice. Supervision of peripheral units can be sporadic because it is not prioritized among competing demands on district management. Traditional management cultures follow hierarchical top-down practices in supervision of auxiliary personnel such as MLHWs, and it is often focused on inspection and fault-finding [8]. In peripheral units where health workers face complex problems with limited resources, non-supportive supervision can even have a detrimental effect on motivation as health workers report feeling that their efforts go unnoticed while mistakes are pointed out immediately [9,10].

A few studies of district-level supervision of health workers in peripheral units have examined the level of implementation of supportive techniques and their association with performance outcomes [11-13]. These studies indicate that key techniques such as problem-solving and developing partnership are implemented at low levels, but are well received by health workers and can contribute to increased productivity and health worker motivation. However, while a few existing studies provide insight into the activities carried out in practice and the potential effectiveness of a supportive approach, the question of *how* supervision can be better implemented at the district level remains unaddressed. To facilitate the translation of recommendations to more supportive practice, there is a need for research that deepens understanding of the process that connects the activities of supervision to health worker performance.

This study examines the operation of health post supervision in rural districts of Guatemala in order to understand the mechanisms by which it contributes to the performance of auxiliary nurses, a cadre of MLHWs. A multiple case study was conducted in five health posts based in principles of realist evaluation. This paper presents a descriptive account of the implementation of supervision of auxiliary nurses in Guatemala, highlighting variation across cases, and an analysis of the mechanisms underlying key differences observed. The insights gained through analysis of the cases are used to refine the program theory that guided the study and offer recommendations for how implementation of MLHW supervision can be strengthened.

## Methods

### Study setting

The study was conducted in the department of Alta Verapaz located in northern Guatemala. The department’s population of 1.1 million is predominantly rural, 90% is indigenous, and it has the highest rate of extreme poverty (38%) and second highest rate of illiteracy (40%) in the country [14]. Pneumonia, acute diarrheal diseases, and malnutrition are among the leading causes of mortality, and maternal mortality is a priority health problem. Health services are provided primarily through the public health system which is overseen at the departmental level by the Regional Health Office in Cobán (the department’s capital), and managed at the municipal level in 19 District Health Offices. The urban seats of the municipalities are served by health centers, and there are two district hospitals and one regional hospital that receive referrals. At the community level, primary care services are provided through health posts and the Coverage Extension Program. Health posts tend to be located in larger villages or clusters of villages, while the Coverage Extension Program covers the most disperse and remote rural population using mobile health teams.

Health posts cover a catchment area of around 2000 inhabitants and are typically staffed by two auxiliary nurses (ANs). Auxiliary nurses are mid-level health workers with one year of training provided through ministry-accredited institutions. A lower entry-level education requirement makes the training more accessible than medicine or professional nursing for indigenous rural residents, thus many ANs in primary care services in Alta Verapaz share the culture and language of the population served. In the health posts, ANs provide basic maternal and child health services, attend consultations, detect and provide treatment for diseases and injuries of low complexity, provide health education in the community, and maintain census and epidemiological data for their catchment area. Their work with maternal and child health services, and specific diseases, such as tuberculosis, is structured by ministry programs which specify guidelines for service delivery and documentation, and indicators for coverage goals of priority services.

Other actors involved in supporting the ANs’ work at the health post level include local leaders, community health volunteers, and additional staff. In order to gain support for their activities and for implementation of health promotion strategies, the ANs’ work depends on communication with local leaders, as well as training of and coordination with health volunteers who have roles specified by the health system. In some health posts, there is also additional staff, including an educator, who helps with health promotion and/or a rotating medical student who attends patients visiting the health post, usually with the AN serving as interpreter.

The health post is connected to the district level through the institutional intervention of supervision. Supervision of ANs is conducted by professional nurses with three-year technical degrees or five-year bachelor degrees, and their training includes some focus on their role in managing care by ANs. At the time of this study (2010), district management included a dedicated primary care supervisor who was in charge of the health posts, in addition to the district nurse manager, and district director. Supervision of the health post includes functions of monitoring completion of the standards of care and production in the ministry programs, developing continuing education and providing accompaniment in activities that promote execution of ministry programs, along with other administrative tasks in the health center. The number of health posts that the supervisor is responsible for varies by district, and in this study it ranged from three to eight.

### Study design

Case studies provide a rigorous methodological path for investigating a phenomenon in its real-life context, and multiple case studies provide the opportunity to examine variations in the phenomenon of interest in different settings [15]. In this study the phenomenon in focus is supervision of auxiliary nurses in rural health posts. In order to better understand how supervision contributes to health worker performance, a realist evaluation approach was applied [16]. Realist evaluation is a theory-driven approach to evaluation, focused on identifying, testing and refining the logic of how an intervention leads to a particular outcome. The logic is articulated in theoretical propositions, which describe the mechanisms of how intervention activities generate outcomes and in which conditions [16]. Because supervision is a social intervention, its operation depends on “mechanisms” located in the participants, that is, how they interpret and act upon or respond to intervention activities [17]. Realist evaluation is a good fit with case study methodology because its focus on mechanisms, outcomes and conditions provide a structure for approaching theory development, which is an essential part of the design phase of case studies [18].

The first step of the process was to formulate a program theory articulating the mechanisms of how the activities of supervision contribute to supporting AN performance based on the models that local actors use implicitly to make sense of their actions [19]. Realist evaluation typically starts from a preliminary middle range theory which draws on existing knowledge and theory to articulate the links between outcomes, mechanisms and context elements. However, program theory can serve as an alternative starting point when little is published or known about the intervention under study, and in this case, published knowledge of how supervision of rural peripheral health units in LMIC health systems operates at the inter-personal level is limited [20]. A program theory articulates the set of assumptions, often held implicitly by the designers and implementers of an intervention, that explain the choice and design of the activities of an intervention and how they are intended to contribute to the desired outcomes [21]. The content of the program theory was developed through the first author’s observations of the organization of supervision of ANs in this setting from previous field work, and was refined through discussion with Guatemalan nurses working in the setting [22]. It describes three central aspects of the supervisor role and how they are intended to influence AN’s motivation and ability to perform:

Regular monitoring motivates the AN to complete their responsibilities because they know that their work is being observed. Individualized support through guidance in problem resolution and fortifying deficient areas improves the AN’s ability to work in the desired way. Accompaniment in work tasks motivates the AN through the interpersonal relationship which allows the AN to feel the support of their supervisor and recognition of their work.

The second step was developing a case study protocol to guide data collection in the health posts. The protocol was elaborated to gather information about what r*egular monitoring, individualized support* and *accompaniment* consisted in and how they were working from multiple sources, including documentation of supervisor visits and interviews with the supervisor and AN. Formulation of guides for interviews and review of documents attempted to capture observable implications of the program theory, or what we could expect to see in the real world if supervision operates as described in the theory. The protocol for data collection also included information about contextual factors that might influence supervision outcomes, including physical conditions and community members’ involvement with the health post.

Selection of cases was the third step. Case selection was based on capturing variance in performance, so that supervision could be observed in health posts with both “strong” and “weak” performance scores. Performance of all health posts in Alta Verapaz was assessed through an efficiency analysis based in data obtained from the Health Management Information System (SIGSA) reflecting outputs in five areas of maternal and child health service delivery and from the human resources records estimating the number of health workers employed during the years 2008, 2009 and 2010. The efficiency of the health posts was analyzed using Data Envelopment Analysis, resulting in scores from zero to 100%, where 100% represents the highest level of efficiency within the sample [23]. Overall, 34 health posts in Alta Verapaz were included in the efficiency analysis used for case selection. Of these, eight had scores over 90% and three had scores under 75% for all of the three years analyzed [24]. In order to gain another view of performance, the results of personnel evaluations conducted in the region by the Units of Nursing and Human Resources in 2009 were also considered in case selection. Together these scores were used to identify health posts likely to have varying conditions influencing performance so that we could observe the operation of supervision in diverse settings.

Replication of case studies provides the opportunity to test and refine the original theoretical proposition [18]. We anticipated that five health post cases selected to represent different regions of Alta Verapaz and different levels of performance would be sufficient to gain insight into the adequacy of the program theory for understanding supervision’s contribution to AN’s motivation and ability to perform in this setting. We selected three health posts with high scores (HP 1 – 3) and two with low scores (HP 4 – 5) from districts in the northeast, southwest and center of the health region as cases. The performance scores used to select these five health posts as well as their characteristics are presented in Table 1.

**Table 1:** Characteristics of health posts

**Health post**	**Performance scores**	**Staffing**	**Population**	**Physical conditions**
***Distance and access to health center***	***TE (2008, 2009, 2010)***^***A***^	***Number and position***	***Dispersion of population covered***	***Services available***
	***AN evaluation***^***B***^	***For ANs: (M/F, years experience)***		***Structural problems reported***
	***HR evaluation***^***C***^			
**HP 1**				
1 hour from HC in microbus (3-4×/day)	TE (100%, 100%, 100%)	2 ANs	Concentrated in 2 communities, within 30 min walking	Potable water
	AN evaluation (NA)	(M, 6 yrs; F, 2 yrs*)		Electricity
	HR evaluation (87%)	1 Educator, part-time		Cellular signal
**HP 2**				
45 min from HC in microbus (every 30 min) and 15 min walking	TE (100%, 100%, 100%)	2 ANs	Disperse – covers 10 communities, most within 30 min, and farthest village 2 hours walking	Rainwater collected
	AN evaluation (80%)	(F, 1.5 yrs*; M, 1 yr)		Electricity
	HR evaluation (80%)	1 Educator, part-time		No cellular signal
		1 Medical student		Leaking roof
				Wall damaged
**HP 3**				
15 min from HC in bus (on main highway, frequent)	TE (100%, 100%, 91%)	2 ANs	Concentrated in 3 communities, all within 15 minutes walking	Potable water
	AN evaluation (81%)	(F, 13 yrs; F, 2 yrs*)		Electricity
	HR evaluation (80%)	1 Educator		Cellular signal
		1 Medical student		Leaking roof
				Wall damaged
**HP 4**				
30 min from HC in microbus (every 30 min)	TE (38%, 46%, 52%)	2 ANs	Concentrated in 1 community within 30 min walking	Rainwater collected
	AN evaluation (NA)	(M, 6 yrs*; F, 2 yrs)		Electricity
	HR evaluation (59%)	1 Educator		Cellular signal
		1 Medical student		
**HP 5**				
30 min from HC in collective taxi (1-2×/day) or 3 hours walking	TE (68%, 48%, 74%)	1 AN (M, 1 yr*)	Disperse – covers 4 communities. Farthest village 1 hr 15 min walking	Potable water
	AN evaluation (61%)	1 Educator		Electricity not connected
	HR evaluation (NA)	1 Medical student		Cellular signal nearby

HP = Health post, HC = Health center, TE = Technical efficiency, AN = Auxiliary nurse, HR = Human resources, NA = Not available.

*AN interviewed. ^A^Technical efficiency scores, Data Envelopment Analysis of secondary data from Regional Health Office [24]. ^B^Evaluation of Auxiliary Nurse Work Performance, Nursing Unit, Regional Health Office, 2009. ^C^Evaluation of Personnel Performance, Human Resources Unit, Regional Health Office, 2009.

### Data collection

The study protocol was reviewed and authorized by the head of the Nursing Unit and the director of the Regional Health Office. Close contact with regional ministry stakeholders in the planning and implementation of the study helped ensure local norms were respected. In line with guidelines of the Guatemalan medical association, ethical clearance was not required as our study did not involve clinical trials or human testing (L. Charnaud, personal communication, Bioethics Committee).

Site visits to the five selected health posts were carried out from November to December 2010 by the first author. The visits were arranged by first contacting the nurse supervisors to inform them about the study, arrange a date to interview them and obtain the telephone numbers of the ANs. The first author then contacted the ANs to explain the nature of the study and set up a date for the visit to the health post. The ANs were asked to arrange for community members who supported the health post to participate in an interview the day of the site visit as well. A total of 14 interviews were conducted, with one AN, one community group and one supervisor interview from each site except for two health posts (HP 3 and 5) that shared a supervisor. Only one of the two ANs on staff was available for interview per site due to the fact that they had begun scheduled vacation for the holidays. The interviews with the supervisors and ANs were conducted in Spanish, and the interviews with the community members were conducted in Spanish with interpreters for Q’eqchi’ and Poqomchi’. Before the interviews, the purpose of the study, the voluntary nature of participation, and procedures for handling the data and protecting confidentiality were reviewed and permission to record was obtained.

The interviews lasted from 20 minutes to one hour, and included open-ended questions to the supervisors and ANs about what supervision consisted in, how activities were perceived to function, how the activities influenced the ANs, as well as questions to ANs about their motivation and their perceptions of their work. The interviews with the community members explored the ANs’ relationship with the community and the nature of community involvement in supporting the health post.

At each health post, the AN provided access to the Register of Activities, records from supervisor visits, and correspondence received. The availability of documents and the extent of documentation varied from site to site. Notes from the review of documents in the health post reflecting contact with the community through health promotion activities, home visits, training for volunteers, meetings with community leaders as well as issues raised during supervisor visits and notifications sent by the ministry during 2009-2010 and other observations were recorded in field notes. Initial analytical impressions of important characteristics of the cases were also recorded after each site visit.

### Data analysis

The interviews were transcribed verbatim and the first stage of analysis focused on writing case reports for each of the health posts, following the strategies proposed by Yin of “relying on theoretical propositions” and “developing a case description” [18]. An index of themes was developed to organize the data from different sources related to the operation of supervision activities described in the initial program theory, as well as characteristics of relevant actors and the context of the health post. The themes were applied to the interview transcripts using NVivo 8.0 software, applied to field notes by hand, and analyzed on a case-by-case basis. Case reports were prepared by the first author which provided a thorough orientation to the health post context, including the physical conditions, the background and characteristics of the ANs and the supervisor, community involvement with the health post, as well as impressions of performance based in activities reflected in the register and observations. Reports also provided detailed description of the activities and outcomes of supervision, including extensive quotes from supervisors and ANs, and initial reflection on mechanisms and the appropriateness of the program theory. The research team reviewed each case report as it was produced. Inclusion of extensive quotes and description provided a basis for discussion around the interpretation of the nature of supervision as well as the influence of contextual factors on performance in each case.

The second stage of analysis focused on examining patterns across cases. Based on iterative review of the reports, the research team identified common and unique characteristics of supervision, and points of interest for further analysis. To facilitate comparison, text from the case reports was sorted into the three categories of supervision activities (monitoring, individualized support and accompaniment). Analysis focused on capturing patterns of similarity and variation in the implementation of supervision activities and their outcomes, uncovering indications of the mechanisms underlying the activities that explain *how* they contributed to the different outcomes observed, and understanding the influence of context on the process of supervision and its outcomes. The account of the operation of supervision articulated in the program theory was then reexamined and the theory was revised in light of the empirical findings.

## Results

The characteristics of the health posts are presented in Table 1. All health posts were located within one hour travel from the local health center, though availability of transportation varied. Staffing included one to two ANs and among those interviewed, three were female and two were male and years of experience ranged from one to six. The dispersion of the population covered varied among health posts with both high and low performance scores. Though most of the communities covered were within 30 minutes walking, HP 2 and 5 included villages that were more than one hour’s walking distance. Regarding physical conditions, the services available (water, electricity, cellular signal) were also variable. Only HP 2 and 3 reported current structural problems (roof and wall damage), while ANs at the other health posts recounted successful efforts to mobilize community support to improve physical conditions.

The basic activities of monitoring and individualized support were similar across the five cases. Monitoring was accomplished through supervision visits as well as district meetings and written reports, which provided a means of evaluating the physical state of the health post, inventory of medicines and supplies, completion of paperwork, progress towards coverage goals, production of services and timeliness of epidemiological data. The activities of individualized support included directing the ANs’ attention to deficient areas and discussing problem resolution, based on the needs detected through monitoring. An important difference emerged in the health posts’ experience of accompaniment. According to all supervisors, accompaniment was an important aspect of their work and it consisted in working beside the AN, being with them while they were working, or “being immersed” in the activities of the health post. However, ANs in HP 2 – 5 reported that they did not receive accompaniment. The AN in HP 1 was unique in reporting that she did receive accompaniment and that she felt her supervisor valued her work.

Even while the activities that supervisors reported they performed across cases were similar, further analysis of supervision in HP 1 indicated that it was *how* she viewed her role that distinguished the case. This led to the identification of the mechanism of supervisors’ orientation as the key factor shaping the implementation of supervision activities and their impact. The mechanism consisted in supervisors’ interpretations of the purpose of their actions in relation to the health system and the health worker, and was manifested in two orientations among supervisors in this study: managerial control (HP 2 – 5) and humanized support (HP 1). The findings are organized below to describe how each of these mechanisms shaped the implementation of the activities of monitoring, individualized support and accompaniment and their outcome. These findings provide the base for revisiting and revising the program theory which guided the study.

### Monitoring

Standard activities of monitoring provided a structure for regular contact between the supervisors and the ANs. The number of supervision visits in the past year varied across the health posts, ranging from two to five visits and lasted a half or full working day. Auxiliary nurses also had contact with their supervisors at least two times a month when turning in reports and picking up supplies at the health center and in monthly district meetings. Supervisors’ focus in implementing these activities was guided by their orientation.

#### Managerial control

For supervisors oriented by managerial control, the primary aim of monitoring was to ensure the completion of ministry guidelines. They approached their role through the framework of the standards and indicators used to evaluate implementation of ministry programs, and their actions aimed to detect deficiencies and motivate the AN to meet these standards. The supervisors’ view of the AN was also closely related to how they interpreted their role and carried out activities. Supervisors expressed that deficits in health post performance were due to lack of motivation among the ANs, who had the “human tendency to neglect certain areas of their work” (Supervisor HP 3 & 5). The controlling function of monitoring was intended to keep ANs focused on completing their responsibilities related to the ministry’s health programs, because “if there were no supervision the auxiliary nurses would stop doing many things that would affect the programs we implement” (Supervisor HP 2).

The outcome of monitoring was seen in the ANs’ motivation to meet the institutional priorities emphasized by their supervisors. The knowledge of what aspects of their work were evaluated was reflected in their own priorities. Even with fewer health post visits, the other monitoring activities oriented the ANs to completing the institutional requirements and these played a prominent role in how they conducted and thought about their work. Awareness of these criteria did motivate the ANs to meet expectations and some expressed that presenting the coverage levels they achieved was a great source of satisfaction in their work.

However, completion of monitoring activities was also a source of stress and pressure from their supervisors: “They tell you, I need this report on this day… And that day you have to see how you are going to get it done… And if you don’t turn it in on time, they start reprimanding you” (AN HP 4). The ANs felt that particularly demands around reporting competed with the time they had for attending patients and the other functions that they had to carry out at the health post, such as home visits and coordination with local leaders and volunteers.

#### Humanized support

The HP 1 supervisor described reviewing progress in the different programs, but she was unique in emphasizing that through monitoring activities she would also find out the ANs’ needs. The supervisor valued the ANs’ assessment of their own situation as important information to guide the actions she took. Her attentiveness to the ANs’ needs was reflected in how she thought about the monthly district meeting:

*“In the (meeting), we see the situation of each health post. We see how they (the ANs) are, what they are missing, what they need, how they want us to help them, or any problem they may have”* (Supervisor HP 1).

The implementation of monitoring activities through the mechanism of humanized support depended on the supervisor’s view of the AN. While in managerial control, supervisors attributed deficits in performance to the ANs’ tendency to neglect, the supervisor of HP 1 stated: “The truth is, their work is hard and they are often alone.” She described that her approach was to congratulate them on the things they are doing well and offer help and encouragement. She acknowledged that ANs had a life larger than their work and suggested that insight into their integral well-being was essential for providing support:

*“Remember that sometimes, since we are human beings, anything can happen to us. Even if we are close by, if we don’t know how they are we can’t support them*” (Supervisor, HP 1).

The idea that you cannot support someone if you do not know how they are reflected that her concept of support was more than a means of correcting deficits in the health post work activities. Rather support was focused on the person performing the work activities.

### Individualized support

The information gathered and the criteria evaluated through monitoring guided the actions that supervisors took to provide individualized support.

#### Managerial control

In HP 2 – 5, individualized support focused primarily on completing reports correctly, meeting coverage goals and correcting problems at the health post, such as lack of materials or physical conditions. Regarding coverage goals, ANs were encouraged to develop action plans to improve coverage, and the supervisors would help them generate strategies. When there were problems with health post conditions, supervisors oriented ANs to the need to solicit outside support, primarily from the municipal government and community leaders. The level of supervisors’ personal engagement in providing support varied across health posts. Some supervisors engaged themselves more directly by assisting the ANs in actions to address the detected problem, such as performing house-to-house sweeps to identify unvaccinated children. The supervisor in HP 4 engaged herself the least, and described that her role was to pass information about problems to the director of the district.

These actions reflected that the standards and criteria for the ministry programs were also central to the supervisors’ approach to individualized support for the ANs in HP 2 – 5. They guided their actions to motivate and in some cases assist ANs to improve their work on the basis of their vision of the desired outcome of supervision. One supervisor illustrated the prominence of this framework in her approach to her role as she named “reaching the percentage of the coverage indicators that the ministry asks for” as the benefit that ANs received from supervision (Supervisor HP 3 & 5).

The outcome of individualized support was evaluated through the ANs’ perception of the benefits they obtained from supervision. Auxiliary nurses in all health posts studied felt they receive support from their supervisors, particularly in the form of help to be a better AN and improve the health post service. However, even though they felt supported, ANs from the two health posts with low performance scores (HP 4 and 5) expressed that their supervisors did not understand their needs. In the view of the AN from HP 5, there was a lack of attention to his learning needs as a recent graduate. He was also working at the most remote health post among the five, and was the only AN working without the support of another AN, so it is possible that his needs for individualized support were greater. In the case of the AN from HP 4, the nature of the needs he expressed were similar to those of other health posts, however, the supervisor’s level of personal engagement in problem resolution was lowest among the cases.

#### Humanized support

The focus of the individualized support given in HP 1 was different from that described in the other health posts. While this supervisor was also checking on ministry programs, her vision of the desired outcome of supervision was *“better care for patients”.* Her focus on patient care was embodied in her actions to provide individualized support, and indicated that she saw her role in the health system not only as manager but also as nurse. There was no mention of problems related to coverage goals. Instead examples of support revolved around patient care and community health issues. The problems that the AN described expressing to the supervisor were related to uncertainties about patient care and concerns about lacking medicines in particular cases. In response, they would review patient records together and the supervisor would try to obtain the medicines at the district hospital.

The supervisor of HP 1 approached the provision of individualized support with a high level of personal engagement. She actively pursued the resolution of problems or addressing needs she detected, and did not just support ANs in resolving their own problems. After the ANs tell her their needs, she described that: “I try to resolve them. If I can, I do so right away. If I can’t, then I find out who can help them resolve their problems” (Supervisor HP 1).

The supervisor’s personal engagement in taking actions to help ANs with their needs was also reflected in the AN’s own actions to solve problems. She described taking initiative to solicit resources to support patients and the work of the health post from various sources. It seemed that the AN benefited not only from the direct support but also from the example set by her supervisor. The ANs had also been successful in mobilizing community support to assist in maintaining good physical conditions at HP 1.

### Accompaniment

The exact activities of accompaniment were difficult to capture because the ANs and supervisors perceived its realization differently. This difference in interpretation highlights that accompaniment is developed in the context of the interpersonal relationship and thus depends on how they view each other.

#### Managerial control

According to supervisors, accompaniment was a prominent part of their approach to their role. Examples given by supervisors included: attending patients who were waiting during a supervisor visit, going out with the AN on a home visit, or filling in reports with them. One supervisor explained that she provided accompaniment “so that the ANs would see supervision as supportive and not only focused on evaluation and fault-finding” (Supervisor HP 3 and 5). However, accompaniment was not part of ANs’ view of supervision: “She just comes to the health post to see what is missing, what is there, or to improve something. That’s what she does. She doesn’t really accompany us” (AN HP 5). These quotes illustrate how ANs and supervisors can view actions intended to provide accompaniment differently.

The expected outcome of accompaniment was ANs feeling the support of their supervisor and recognition of their work and efforts. While the ANs did feel supported through specific actions, they expressed that supervisors did not understand the demanding nature of their day-to-day work attending patients and maintaining the health post. Since their efforts were not recognized, ANs from HP 2 – 5 felt that supervisors did not value their work, which is illustrated in the following quotes.

*“[The supervisor] doesn’t work with us. She doesn’t realize what we do… She thinks that we don’t do anything, when we are really putting ourselves out to make sure that everything [at the health post] is okay…”* (AN HP 2).

*“Here we provide a thousand services, as janitor, doctor, midwife… we suture, see patients, give nutrition supplements, vaccinate, so many things. In this sense, they have a hard time understanding us… Now there is so much paperwork…We are human beings and we have limits”* (AN HP 4).

The ANs’ perceptions that their supervisors did not understand the full scope of the demands they faced and even thought that they “don’t do anything” reflect how the managerial control orientation influenced the ANs’ experience of the interpersonal relationship. Despite supervisors’ efforts to accompany the ANs in their work activities, their focus on the goals of “meeting the percentage of the indicators that the Ministry asks for” meant that recognition of the ANs’ work was limited to their actions to meet and document these criteria. However, in the health post, the ANs also experienced other pressures:

*“Here the people are the ones who demand the most from us because they need us… And what the people value the most is the support that they feel from us”* (AN HP 2).

#### Humanized support

Accompaniment was perceived to be part of supervision by both supervisor and AN in HP 1. However, as seen in the other health posts the actions of working beside the AN and assisting them with tasks were not in themselves seen as accompaniment. Instead, the realization of accompaniment in HP 1 was infused in the other activities of supervision through attention to the ANs’ needs, and active engagement in addressing these needs.

The outcome of these activities was seen in the AN’s sense that the supervisor valued the work of the health post, which contrasted with the feeling of ANs from the other health posts. The AN explained that she knew the supervisor valued their work because she recognized their efforts in patient care and complimented them on how they handle their many roles in the health post. Recognition of the nature of the ANs’ work, combined with attention to their needs and active engagement in problem-solving seemed to form an integral basis for development of a supportive interpersonal relationship that enabled the AN’s motivation and ability to perform.

The supervisor’s orientation towards “better patient care” in her role provided grounds for a shared sense of value with the ANs regarding the importance of their work. The AN from HP 1 expressed that the most important and satisfying aspect of her work is “to fulfill the need that the people have” and help them feel better. ANs from the other two well-performing health posts (HP 2 and 3) also reflected this orientation to nursing care and their relationship with the patients in their sense of the importance of their work, even though it did not seem to be emphasized by their supervisors.

### Revisiting the program theory

**Figure 1:**
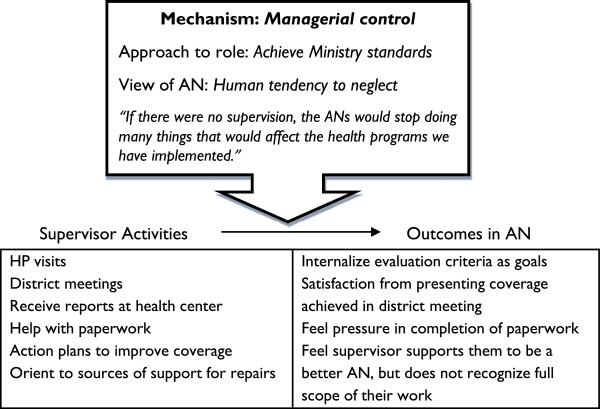
A managerial control approach to supervision activities and their outcomes.

**Figure 2:**
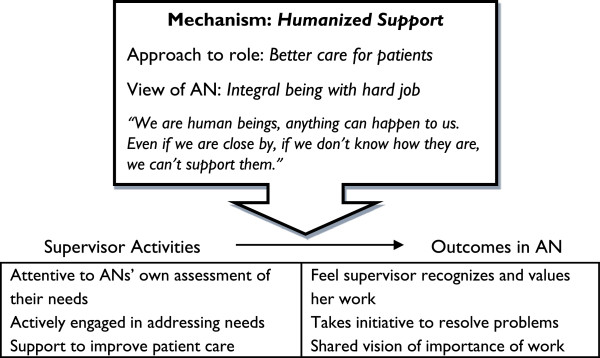
A humanized support approach to supervision activities and their outcomes.

In the original program theory that guided the study, supervision was described as a set of activities intended to generate outcomes in health workers that help improve their performance. The variation observed in the way supervision was implemented suggested that these activities cannot be understood as a checklist of techniques. Attention to the manner in which supervisors interpreted their role and their view of the AN revealed patterns that were consistent with the variation seen in their activities and the outcomes in the ANs across health post cases (see Figures 1 and 2).

The description of monitoring and individualized support provided in the original program theory was mostly consistent with operation of supervision in health posts 2 – 5. However, the description of the outcomes as “completing responsibilities” and “improved ability to work in the desired way” reflected an assumption that there was a shared view of what constituted good performance. While the supervisors of health posts 2 – 5 indicated that their view of performance was based in implementation of ministry programs, the supervisor of health post 1 and the ANs from well-performing health posts also highlighted the importance of nursing care in their work. In this way, the supervisor’s own interpretation of priorities influenced the focus of their activities.

Based on these findings, we revised the program theory as follows to reflect that the way supervision activities are implemented and their outcomes depend on the mechanism of how the supervisor approaches her role and how she views the AN.

Monitoring orients ANs’ work to the responsibilities that supervisors prioritize and individualized support helps ANs to attain them in the desired way. Accompaniment develops a supportive relationship with the AN through attention and response to their needs. Conditions orienting supervisors’ approach to their role and their relationship to the AN include the institutional guidelines for implementing and evaluating ministry programs and the vocation and humanistic values of the nursing profession. Depending on the supervisors’ dominant orientation, the outcomes of supervision can be seen in ANs’ motivation and ability to complete institutional guidelines and also a fortified sense of the value of their work attending patient needs.

## Discussion

This study showed how the implementation of supervision was shaped by the mechanism of supervisors’ approach to their role and view of the health worker. The mechanisms of managerial control and humanized support generated different responses in the ANs, illustrating how supervisors’ orientation affects the nature of the support they provide to the health worker and their potential contribution to health workers’ performance.

Managerial control was the dominant mechanism of health post supervision, and the orientation towards a top down application of standardized criteria is consistent with the characterization of rural supervision visits in other studies [3,10,25]. This approach reflected the priorities of the ministry’s institutional culture, which is strongly influenced by the standardized procedures and criteria used to evaluate the performance of health service units. Efforts to be supportive focused on “supporting” ANs to attain these standards. The view of the AN as negligent and in need of control also reflected a hierarchical organizational culture, where supervisors see themselves in a position of authority over auxiliary personnel. In response, ANs reflected their supervisors’ orientation to ministry criteria and expressed that they received some support but felt the full scope of their work in the health post was not recognized.

The mechanism of humanized support was present in the case of HP 1 and was closely related to the supervisor’s professional identity as a nurse. She voiced concerns and described efforts for improving care that reflected her vocation and professional experience. Her view of the AN as an integral human being and her attentiveness to their own perceptions of their needs reflect the nature of the interpersonal relationship that forms the basis for nursing practice [26,27]. Though in this case the interpersonal relationship is between a nurse supervisor and an auxiliary nurse, the parallels between nurse-patient and supervisory relationships have been elaborated elsewhere [28-30]. Support was oriented to the person in the health worker role and the process of improving patient care, and the outcome was seen in the AN’s sense that the supervisor valued her work and their shared vision of the importance of patient care.

Supervision in HP 1 mirrored recommendations for supportive supervision which call for more focus on patient care issues and resolution of problems experienced by health workers and less emphasis on facility inspection and service statistics [7]. Understanding the influences shaping the different approaches observed in this study can indicate directions for strengthening implementation of supportive supervision. The cases of HP 2 – 5 illustrated that even while supervisors recognized the value of providing support through accompaniment, the ministry’s standardized criteria for assessing health post performance directed emphasis to inspection and statistics. Focus on improving patient care was in line with the ministry’s vision of “providing comprehensive service with quality and care”, however this goal had not been operationalized in guidelines for supervision. In HP 1, the supervisor’s approach integrated principles from the nursing profession in her orientation to performance goals and her relationship to the AN. The operation of supervision in this case indicated that professional ethos provided the base for a more supportive implementation through focus on patient care and attunement to ANs’ integral needs. While other studies have emphasized the need for managerial training to fortify implementation of supportive supervision, this study suggests that promoting supervisors’ professional ethos can contribute to strengthened support for health workers [12,31,32]. This strategy is complementary to findings of other studies that point to sense of vocation as an important motivating factor for rural health workers [10,22,25,33].

Existing organizational configurations in health systems play an important role in how interventions to support performance are implemented, and understanding how supervisors related to institutional cultures in this context provides a basis for considering the transferability of the findings [8]. Promoting nursing principles of care as means of developing supervisors’ orientation to humanized support may be particularly relevant for other low and middle-income country settings where nurses are responsible for supervision of peripheral health units, and cadres of MLHWs are formed within the discipline of nursing. The findings may also be useful more generally in indicating a direction for approaching the supervision relationship with MLHWs through professional ethos and promoting a shared vision of improved patient care, particularly in settings where managerial control tendencies predominate.

This study contributed to a deeper understanding of how supervision contributes to health worker motivation and performance in a rural health care setting. However, supervision is one of many factors influencing the motivation and performance of health workers and differences in supervision alone did not account for variation in health posts’ measured performance levels. Attention to other factors shaping performance, such as health worker competence and the community’s care-seeking behavior, would provide further insight into how performance levels were reached. In order to better understand supervision’s contribution, there is a need to follow the process further to understand how the impact of supervisors’ support on AN motivation translates to performance outcomes, and how supervision interacts with other interventions to support health workers, including community participation.

### Methodological considerations

Realist evaluation was a useful tool for analyzing the operation of supervision across cases. The original program theory provided a framework for data analysis, but its description of the mechanisms of the ANs’ expected response to specific supervision activities did not adequately explain the differences observed. Variation in the implementation of supervision seemed to play a more important role. Focusing on mechanisms at this level enabled us to make sense of the patterns by looking beyond the activities themselves to identify the core of the difference in the supervisors’ reasoning. Previous realist studies of human resources management interventions for improving performance have identified mechanisms activated in the health workers (increased knowledge and skills, increased motivation, feeling obliged to change) and at the organizational level (perceived organizational support, reciprocity [19,32]. Bringing the supervisor’s interpretation of her role into focus as a mechanism of the intervention adds important information about how the relationship that connects the peripheral health unit to the health system contributes to the health worker’s experience of support.

Key contextual conditions influencing health post supervision were not specified in the program theory, but were examined in an exploratory manner. Information collected on physical conditions at the health posts revealed a range of challenges related to infrastructure and resource availability, but there was no pattern of influence on how supervision was operating. In fact, we observed that these conditions were also modified through ANs’ initiatives to mobilize support from the community, and thus could also potentially reflect ANs’ motivation and their capacity to shape their context. We found that the institutional context was more important in guiding supervisor behavior, including the structures and culture of the ministry and the principles of the nursing profession. However, there was no clear evidence explaining why the mechanism of humanized support had been triggered in HP 1 while managerial control was active in the others [16]. Further exploration is needed to gain understanding of factors at the individual and interpersonal levels that may influence how the supervisors relate to the institutions in their context. Initial observations indicate that the work environment at the district level and integration of nursing vocation with religious desire to serve others may be important conditions influencing supervisions’ orientation in this setting.

This study analyzed supervision’s effect on AN motivation through the mechanism of the supervisor’s approach to her role. Alternative interpretations of the causal relationship redirect focus to the effect of the ANs’ motivation on the way supervision is carried out. Many supervisors in this setting expressed that lack of AN motivation was the main difficulty they faced in impacting performance. Our assessment of motivation indicated that ANs in the five health posts felt their work was valuable and took initiative to improve conditions in collaboration with the community. In the three “well-performing” health posts, ANs expressed a stronger sense of vocation and valued their capacity to attend patients’ needs. Our understanding of the inter-relationship between supervision and motivation could have been strengthened through repeated contacts, interviews with other personnel in the health posts and district management, and extended observation of practice, if time and resources allowed.

Insight into the contribution of humanized support and professional ethos to implementation of supportive supervision for MLHWs was gained from the operation of supervision in one health post. Identification of HP 1 as a special case was based on cross-case comparison as well as impressions of performance monitoring in the ministry gained through field work. Based on one case, this study cannot offer conclusions regarding the effectiveness of supervision through the humanized support approach. However, the findings from this case provided evidence of a locally-generated orientation that deviates from the dominant institutional norms and reflects principles of supportive supervision. Findings across cases also indicated that the idea of supporting ANs through “accompaniment” is common among supervisors in this setting. These findings can serve as a starting point for further exploration of supervisors’ interpretation of their role and how their managerial style is generated in order to identify aspects that can be fortified to promote implementation of more supportive supervision in this context, or similar contexts.

## Conclusions

This study demonstrated that supportive supervision consists of more than a checklist of techniques. Application of a realist evaluation approach revealed that the influence of supervision activities on health workers’ motivation and ability to perform depended on the mechanism of supervisors’ orientation. In this study the orientation of managerial control predominated. However, the operation of supervision in one health post indicated that shared focus on improving patient care and an integral view of the health worker’s needs provided the grounds for a more humanized, supportive relationship to the AN. Supervisors’ orientations reflected the influence of different institutions in their organizational context, and in this setting, nursing principles were central to a more supportive approach. Efforts to strengthen the support that supervision provides to MLHWs should promote professional ethos as a means of developing shared performance goals and orient supervisors to a more holistic view of the health worker and their work.

## Abbreviations

AN: Auxiliary nurse; HP: Health post; MLHW: Mid-level health worker.

## Competing interests

All authors declare they have no competing interests.

## Authors’ contributions

ARH contributed to the study design, acquisition of data, analysis and manuscript writing. MSS supervised the study process from design to manuscript editing. MSS, AKH, and KD participated in analysis, critically reviewed the manuscript and contributed to intellectual content. All authors read and approved the final manuscript.

## Pre-publication history

The pre-publication history for this paper can be accessed here:

http://www.biomedcentral.com/1472-6963/14/112/prepub

## References

[B1] Lehmann U Mid-Level Health Workers. The State of the Evidence on Programmes, Activities, Costs and Impact on Health Outcomes. A Literature Review 2008 World Health Organization: WHO

[B2] Fonn S Ray S Blaauw D Innovation to improve health care provision and health systems in sub-Saharan Africa - promoting agency in mid-level workers and district managers Glob Public Health 2011 14 657 668 10.1080/17441692.2010.489905 20582782

[B3] Bosch-Capblanch X Garner P Primary health care supervision in developing countries Trop Med Int Health 2008 14 369 383 10.1111/j.1365-3156.2008.02012.x 18397400

[B4] Gilson L Trust in health care: theoretical perspectives and research needs J Health Organ Manag 2006 14 359 375 10.1108/14777260610701768 17087400

[B5] Criel B De Brouwere V Managerial Supervision to Improve Primary Health Care in Low-and Middle-Income Countries: RHL Commentary WHO Reproductive Health Library 2012 Geneva: World Health Organization

[B6] WHO World Health Report 2000: Health Systems - Improving Performance 2000 Geneva: World Health Organization

[B7] Marquez L Kean L Making Supervision Supportive and Sustainable: New Approaches to Old Problems Maximum Access and Quality (MAQ) Initiative Series 2002 Washington DC: Management Sciences for Health

[B8] Blaise P Kegels G A realistic approach to the evaluation of the quality management movement in health care systems: a comparison between European and African contexts based on Mintzberg’s organizational models Int J Health Plann Manag 2004 14 337 364 10.1002/hpm.769 15688877

[B9] Clements J Streefland P Malau C Supervision in primary health care - can it be carried out effectively in developing countries? Curr Drug Saf 2007 14 19 23 10.2174/157488607779315435 18690946

[B10] Mathauer I Imhoff I Health worker motivation in Africa: the role of non-financial incentives and human resource management tools Hum Resour Health 2006 14 24 10.1186/1478-4491-4-24 16939644 PMC1592506

[B11] Abou El Enein NY Mohammed SA Zaghloul AA Health care providers’ views on supervisory visits in family health centers and units in Alexandria, Egypt J Egypt Publ Health Assoc 2010 14 189 204 21244817

[B12] Tavrow P Kim YM Malianga L Measuring the quality of supervisor-provider interactions in health care facilities in Zimbabwe Int J Qual Health Care 2002 14 Suppl 1 57 66 12572788 10.1093/intqhc/14.suppl_1.57

[B13] Frimpong JA Helleringer S Awoonor-Williams JK Yeji F Phillips JF Does supervision improve health worker productivity? Evidence from the Upper East Region of Ghana Trop Med Int Health 2011 14 1225 1233 10.1111/j.1365-3156.2011.02824.x 21729221

[B14] ENCOVI Pobreza y Desarrollo: Un Enfoque Departamental 2011 Guatemala: Instituto Nacional de Estadística

[B15] Stake RE Multiple Case Study Analysis 2006 New York: Guilford Press

[B16] Pawson R Tilley N Realistic evaluation 1997 New York: Sage

[B17] Astbury B Leeuw FL Unpacking black boxes: mechanisms and theory building in evaluation Am J Eval 2010 14 363 381 10.1177/1098214010371972

[B18] Yin RK Case Study Research: Design and Methods 2008 Thousand Oaks: Sage

[B19] Marchal B Dedzo M Kegels G A realist evaluation of the management of a well-performing regional hospital in Ghana BMC Health Serv Res 2010 14 24 10.1186/1472-6963-10-24 20100330 PMC2828434

[B20] Marchal B van Belle S van Olmen J Hoerée T Kegels G Is realist evaluation keeping its promise? A review of published empirical studies in the field of health systems research Evaluation 2012 14 192 212 10.1177/1356389012442444

[B21] Van Belle S Marchal B Dubourg D Kegels G How to develop a theory-driven evaluation design? Lessons learned from an adolescent sexual and reproductive health programme in West Africa BMC Public Health 2010 14 1 10 10.1186/1471-2458-10-1 21118510 PMC3001738

[B22] Hernandez A Hurtig AK Dahlblom K San Sebastian M Translating community connectedness to practice: a qualitative study of midlevel health workers in rural Guatemala ISRN Nursing 2012 14 648769 23097715 10.5402/2012/648769 PMC3477764

[B23] Coelli TJ Rao DSP O’Donnell CJ Battese GE An Introduction to Efficiency and Productivity Analysis 2005 London: Kluwer Academic Publishers

[B24] Hernandez AR San Sebastian M Assessing the technical efficiency of health posts in rural Guatemala: a data envelopment analysis Glob Health Action 2014 14 23190 24461356 10.3402/gha.v7.23190 PMC3901389

[B25] Manongi RN Marchant TC Bygbjerg IC Improving motivation among primary health care workers in Tanzania: a health worker perspective Hum Resour Health 2006 14 6 10.1186/1478-4491-4-6 16522213 PMC1450300

[B26] Gastmans C Interpersonal relations in nursing: a philosophical-ethical analysis of the work of Hildegard E. Peplau J Adv Nurs 1998 14 1312 1319 10.1046/j.1365-2648.1998.00840.x 9888377

[B27] Peplau HE Interpersonal Relations in Nursing 1952 New York: Putnam’s Sons

[B28] Antrobus S Developing the nurse as a knowledge worker in health—learning the artistry of practice J Adv Nurs 2008 14 829 835 10.1046/j.1365-2648.1997.1997025829.x 9104682

[B29] Playle JF Mullarkey K Parallel process in clinical supervision: enhancing learning and providing support Nurse Educ Today 1998 14 558 566 10.1016/S0260-6917(98)80006-3 9887755

[B30] Butterworth T Faugier J Burnard P Clinical Supervision and Mentorship in Nursing 1998 London: Nelson Thornes

[B31] Rowe AK de Savigny D Lanata CF Victora CG How can we achieve and maintain high-quality performance of health workers in low-resource settings? Lancet 2005 14 1026 1035 10.1016/S0140-6736(05)67028-6 16168785

[B32] Dieleman M Gerretsen B Van Der Wilt GJ Human resource management interventions to improve health workers’ performance in low and middle income countries: a realist review Health Res Pol Syst 2009 14 7 10.1186/1478-4505-7-7 PMC2672945 19374734

[B33] Serneels P Montalvo JG Pettersson G Lievens T Butera JD Kidanu A Who wants to work in a rural health post? The role of intrinsic motivation, rural background and faith-based institutions in Ethiopia and Rwanda Bull World Health Organ 2010 14 342 349 10.2471/BLT.09.072728 20461138 PMC2865659

